# Identification of galectin-1 as a novel mediator for chemoresistance in chronic myeloid leukemia cells

**DOI:** 10.18632/oncotarget.8489

**Published:** 2016-03-30

**Authors:** Wu Luo, Li Song, Xi-Lei Chen, Xiang-Feng Zeng, Jian-Zhang Wu, Cai-Rong Zhu, Tao Huang, Xiang-Peng Tan, Xiao-Mian Lin, Qi Yang, Ji-Zhong Wang, Xiao-Kun Li, Xiao-Ping Wu

**Affiliations:** ^1^ Institute of Tissue Transplantation and Immunology, Key Laboratory of Functional Protein Research of Guangdong Higher Education Institutes, Key Laboratory of Molecule Immunology and Antibody Engineering of Guangdong Province, Jinan University, Guangzhou, 510632, China; ^2^ School of Pharmaceutical Science, Wenzhou Medical University, Wenzhou, 325035, China; ^3^ Guangzhou Women and Children's Medical Center, Guangzhou, 510623, China

**Keywords:** galectin-1, chronic myelogenous leukemia, chemoresistance, MDR1, P38 MAPK

## Abstract

Multidrug resistance protein-1 (MDR1) has been proven to be associated with the development of chemoresistance to imatinib (Glivec, STI571) which displays high efficacy in treatment of BCR-ABL-positive chronic myelogenous leukemia (CML). However, the possible mechanisms of MDR1 modulation in the process of the resistance development remain to be defined. Herein, galectin-1 was identified as a candidate modulator of MDR1 by proteomic analysis of a model system of leukemia cell lines with a gradual increase of MDR1 expression and drug resistance. Coincidently, alteration of galectin-1 expression triggers the change of MDR1 expression as well as the resistance to the cytotoxic drugs, suggesting that augment of MDR1 expression engages in galectin-1-mediated chemoresistance. Moreover, we provided the first data showing that NF-κB translocation induced by P38 MAPK activation was responsible for the modulation effect of galectin-1 on MDR1 in the chronic myelogenous leukemia cells. Galectin-1 might be considered as a novel target for combined modality therapy for enhancing the efficacy of CML treatment with imatinib.

## INTRODUCTION

Formation of the *BCR-ABL* fusion gene coding for a constitutively active *BCR-ABL* tyrosine kinase via t(9;22)(q34;q11) reciprocal translocation initiates 95% of chronic myelogenous leukemia (CML), and 25% of adults and 5% of children acute lymphoblastic leukemia (ALL) [1]. Imatinib provides a promising treatment for CML by high selectively binding to the ATP-binding site of *BCR-ABL* and inhibiting *BCR-ABL* activation [2]. However, the occurrence of drug resistance was reported in CML patients with advanced stages treated with imatinib [3]. Amplification of the *BCR-ABL* gene and mutations of the kinase domain of ABL have been described as the molecular mechanisms for the development of imatinib resistance [4-9]. However, overexpression or mutations of *BCR-ABL* could not explain all drug resistance to imatinib in CML patients, implying that the alternative mechanisms may exist [10-12].

MDR1, the *ABCB1* gene product, is an ABC transporter at the cell surface responsible for extruding the compounds out of the cell, and has the potentials of mediating multiple drug resistance (MDR) by reducing intracellular drug concentrations [13]. Previous investigations showed that imatinib is the substrate of MDR1 and considered drug efflux mediated by MDR1 as a causal role for imatinib drug resistance in CML [14, 15]. But the precise mechanisms of MDR1 modulation in chronic myelogenous leukemia cells remain to be unclear. In the current study, we first applied proteomic approach to identify galectin-1 as a candidate of MDR1 modulators for mediating drug resistance in CML cells by comparison of the protein profiles among a model system of leukemia cell lines with a gradual increase of MDR1 expression and drug resistance, and further explored the mechanisms of galectin-1 acting as a novel MDR1 modulator contributing to functional resistance against the cytotoxic drugs.

## RESULTS

### Characterization of the MDR phenotype in K562, K562/ADM and the revertant K562/ADM cells

Initially, the sensitivity profiles against adriamycin and imatinib were explored in a model system of cell lines including K562, K562/ADM and the revertant K562/ADM cells. As shown in Table 1, the resistant cell line K562/ADM displayed higher resistance against adriamycin and imatinib, with 50-fold and 5-fold increase of IC_50_ for adriamycin and imatinib respectively, than its sensitive counterpart K562 cells. We observed that the revertant K562/ADM cell line showed less resistance than K562/ADM cell line but higher resistance than K562 cell line against both adriamycin and imatinib, suggesting the resistant cells gradually lose the resistant character when cultured in the absence of the chemical compound.

**Table 1:** The IC_50_ of adriamycin and imatinib in K562, K562/ADM and the revertant K562/ADM cells

IC_50_	K562	K562/ADM	revertant K562/ADM
ADM(μg/ml)	0.465±0.04	26.56±0.43	17.56±0.53
imatinib(μM)	0.2±0.039	2.3±0.218	1.7±0.12

**Figure 1:**
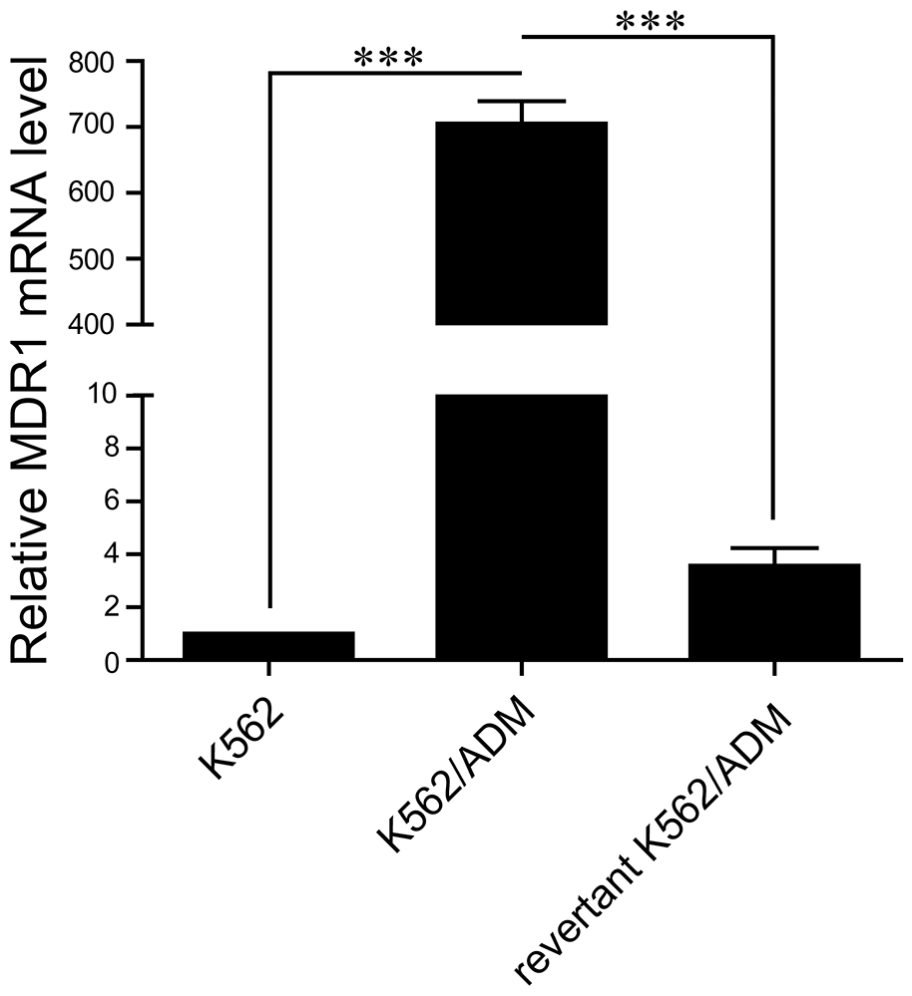
Comparison of MDR1 expressions in three types of K562 cell lines The mRNA levels of MDR1 in K562, K562/ADM, and the revertant K562/ADM cells were detected by q-PCR. Data are presented as the mean (±SD) of three independent experiments. ***p<0.001.

Quantitative PCR (q-PCR) analysis showed that the expression level of MDR1 in the revertant K562/ADM cells is less than the resistant K562/ADM cells, but higher than the sensitive ones, suggesting that the expression level of MDR1 gradually decreases during the course of cultivation in the absence of adriamycin (Figure 1). The results imply that a gradual increase of resistance against adriamycin and imatinib is accompanied by a gradual increase of MDR1 level during the course of development of drug resistance in K562 cells exposed to adriamycin.

### Identification of galectin-1 as a significantly up-regulated protein in resistant K562 cells by 2D-PAGE and MALDI-TOF-TOF mass spectrometry

**Figure 2:**
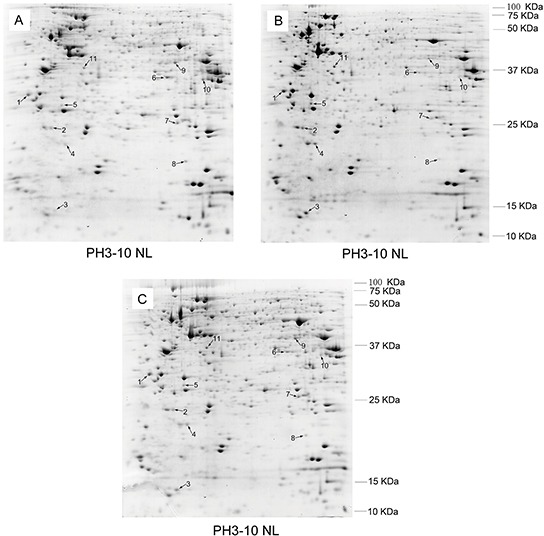
Comparison of the protein expression patterns between K562 **A.** and the revertant K562/ADM cells **B.** and the revertant K562/ADM cells **C.** The proteins were separated by two-dimensional electrophoresis and stained with Coomassie brilliant blue G250. Identified protein spots are indicated by numbers.

In order to elucidate the mechanisms of MDR1 modulation in K562 cells, a proteomic approach was initially applied to identify differentially expressed proteins among three types of K562 cells with different MDR1 expression and potentials of drug resistance. As shown in Figure 2, the well-resolved, reproducible 2D-PAGE patterns of K562 (Figure 2A), K562/ADM (Figure 2B), and the revertant K562/ADM cells (Figure 2C) were established, and yielded about 1000 protein spots each. In total, 11 protein spots were found to be differentially expressed among the investigated cells. All of these were excised and analyzed by MALDI-TOF-TOF mass spectrometry and a subsequent search in the IPI databases for protein identification. The identification information was summarized in Table 2.

**Table 2:** MALDI-TOF-TOF results of the differentially expressed proteins

Spot No.	Protein description	Gene name	Function	Accession no.	Theoretical Mw/pI	Score	C.I.%	Expr. level (K562 vs K562/ADM)
1	Complement component 1 Q subcomponent-binding protein	C1QBP	Cell apoptosis, proliferation and migration	IPI00014230	31741.8/4.74	282	100	+
2	Lactoylglutathione lyase	GLO1	Metabolism	IPI00220766	20992.3/5.12	523	100	+
3	Galectin-1	LGALS1	Cell apoptosis, proliferation and differentiation	IPI00219219	15048.3/5.34	295	100	+
4	Sorcin isoform b	SRI	Calcium-binding protein	IPI00414264	20616.9/ 5.11	150	100	+
5	Isoform 2 of Proteasome subunit alpha type-3	PSMA3	Proteolysis	IPI00171199	27857.8/5.19	349	100	+
6	Isoform 2 of Heterogeneous nuclear ribonucleoprotein D-like	HNRPDL	Cellular transcription	IPI00845282	33739.2/6.85	290	100	-
7	Proteasome subunit alpha type-2	PSMA2	Proteolysis	IPI00219622	25996.3/6.92	259	100	-
8	Isoform 2 of Transcription factor BTF3	BTF3	Cellular transcription	IPI00419473	17688.2/6.85	185	100	-
9	Poly(rC)-binding protein 1	PCBP1	Signal transduction	IPI00016610	37987.1/6.66	526	100	-
10	Isoform A2 Heterogeneous nuclear ribonucleoproteins A2/B1	HNRNPA2B1	Cellular transcription	IPI00414696	36040.9/8.67	714	100	-
11	Eukaryotic translation initiation factor 3 subunit I	EIF3I	Signal transduction	IPI00012795	36478.6/5.38	300	100	+

**Figure 3:**
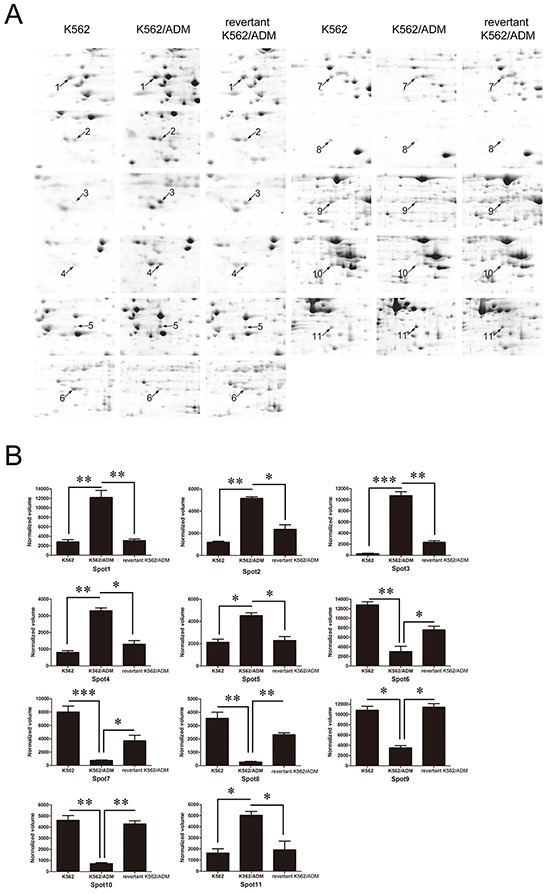
**A.** Enlarged maps of the protein spots differentially expressed among K562, K562/ADM, and the revertant K562/ADM cells. **B.** Graphical representation of spot intensities assigned by PDQuest 8.0 software subsequent to normalization. The graphs show the intensities of the protein spots differentially expressed among K562, K562/ADM, and the revertant K562/ADM cells. *p<0.05, **p<0.01, ***p<0.001.

Among the identified differentially expressed proteins, six of them were significantly up-regulated in K562/ADM cells compared with K562 cells, and significantly down-regulated in the revertant K562/ADM cells compared with K562/ADM cells. Whereas, the expression levels of the other five identified proteins in the revertant K562/ADM cells were less than those in K562 cells, and higher than those in K562/ADM cells (Figure 3). The differentially expressed proteins related to cell behaviors, metabolism, calcium-binding, proteolysis, cellular transcription, and signal transduction maybe valuable for further elucidating the chemoresistant mechanisms in CML.

Among the identified proteins, galectin-1 (Gal-1), which exerts effects on cell apoptosis, proliferation and differentiation, increased 4.85 folds in K562/ADM cells compared with the revertant K562/ADM cells, and up-regulated 22.3 folds compared with K562 cells, implying that galectin-1 may contribute to augment of MDR1 expression and drug resistance in CML.

### Verification of galectin-1 expression

**Figure 4:**
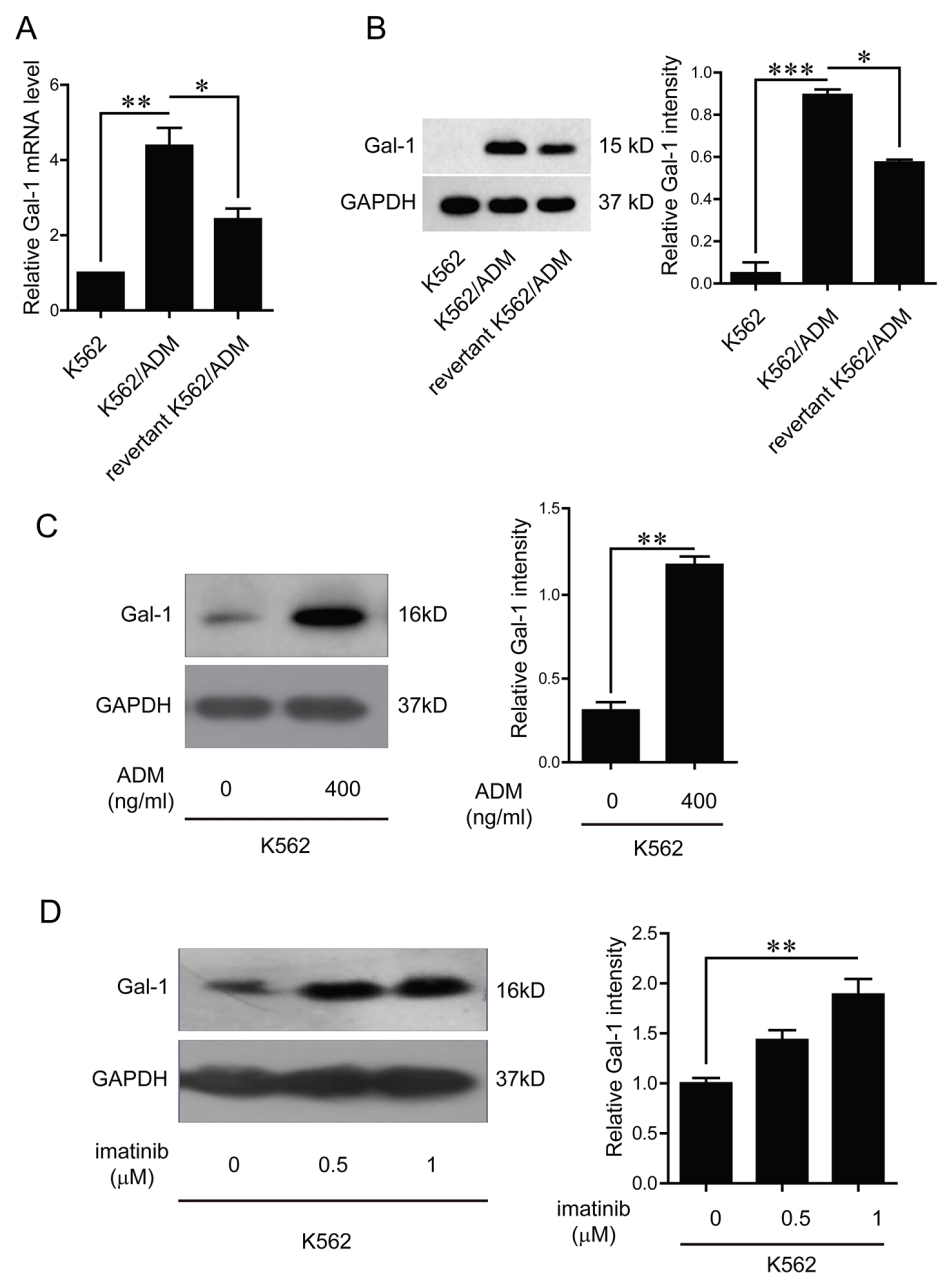
Verification of galectin-1 expression and effects of chemical drugs on galectin-1 product **A.** Total RNA was isolated from K562, K562/ADM, and the revertant K562/ADM cells, and subjected to q-PCR analysis of the mRNA levels of galectin-1. **B.** The protein levels of galectin-1 were detected by western blotting and scanning densitometry. Data are presented as the expression level relative to that of GAPDH. **C.** K562 cells were treated with adriamycin for 48 h before extraction of cell lysates, which were subsequently immunoblotted with galectin-1 antibody. **D.** K562 cells were treated with imitinib for 48 h, and whole cell lysates were subsequently immunoblotted with galectin-1 antibody. Data are presented as the mean (±SD) of three independent experiments. *p<0.05, **p<0.01, ***p<0.001.

In order to confirm the trends of the expression levels of galectin-1 identified by the proteomic approach, q-PCR method was first applied to measure the mRNA levels of galectin-1. The results present in Figure 4A indicated that the mRNA level of galectin-1 in K562/ADM was 1.87 folds higher than the revertant K562/ADM cells, and 4.4 folds than K562 cells, respectively. Further western blot analysis revealed a significant up-regulation of galectin-1 expression in K562/ADM cells compared with the other two types of cells with less drug resistance, which were paralleled to the protein level changes observed in the proteomic analysis (Figure 4B).

As the expression levels of galectin-1 increased with the augment of drug resistance, and the resistant cell lines were selected by exposure to adriamycin, we speculated it may be the chemical drugs that trigger the increase of galectin-1 product. Further investigations indicated that both adriamycin and imatinib up-regulated galectin-1 expression in a dose dependent manner (Figure 4C and 4D).

### Galectin-1 mediates chemoresistance in CML

**Figure 5:**
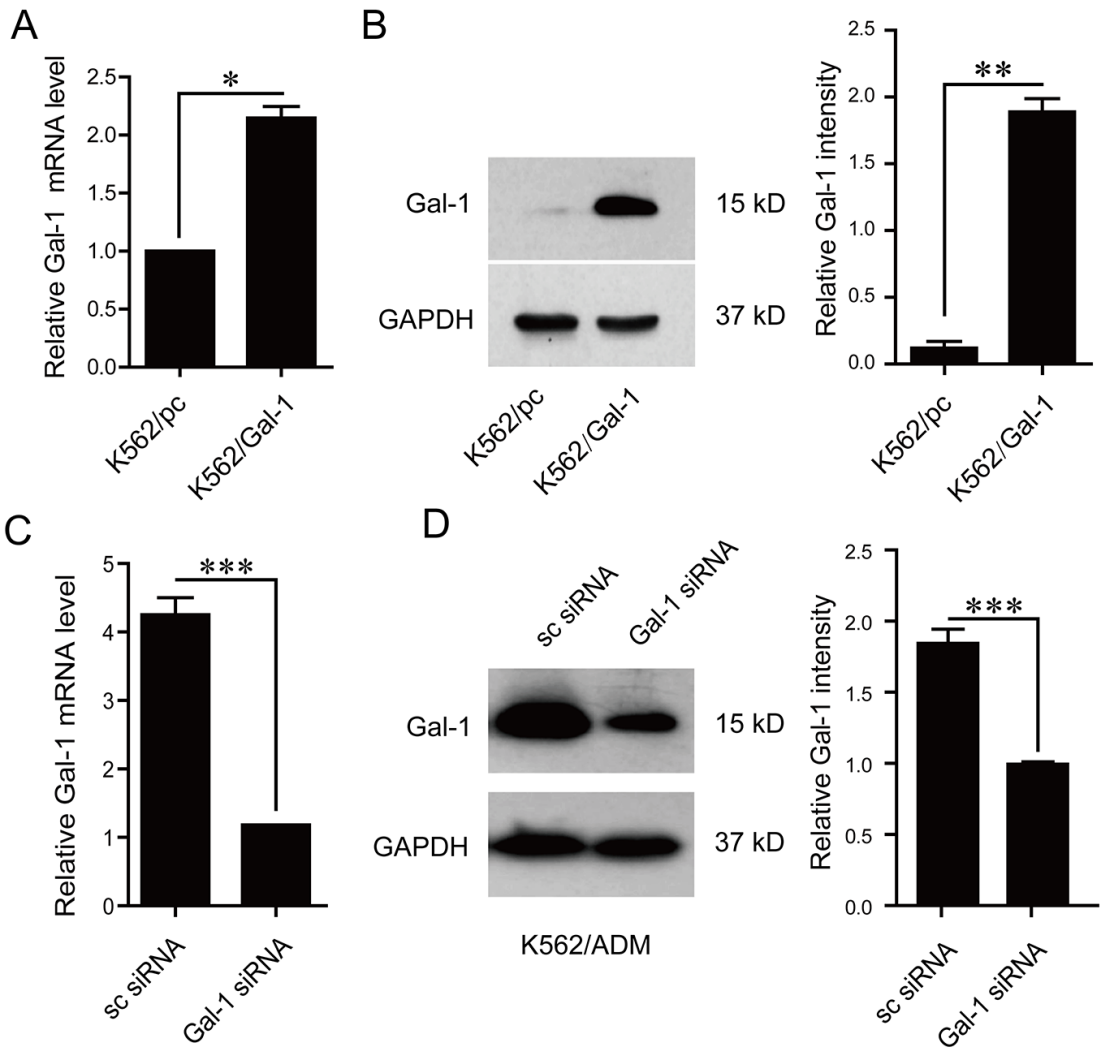
Effects of galectin-1 on drug resistance **A.** The mRNA levels of galectin-1 in K562 cells stably transfected with pcDNA3.1(-) or pcDNA3.1(-)/Gal-1 were assessed by q-PCR. **B.** Increased protein levels of galectin-1 in pcDNA3.1(-)/Gal-1 stably transfected K562 cells (K562/Gal-1) were determined in whole cell lysates by western blotting. **C.** K562/ADM cells were transfected with galectin-1 targeting siRNA (Gal-1 siRNA) or scrambled siRNA (sc siRNA) for 48 h prior to analysis of galectin-1 mRNA levels by q-PCR. **D.** Decreased protein levels of galectin-1 in Gal-1 siRNA transfected K562/ADM cells were detected using western blotting. MTT assay was applied to determine the effects of alteration of galectin-1 expression on the resistance to adrimycin and imatinib as present in Table 3. Data are presented as the mean (±SD) of three independent experiments. *p<0.05, **p<0.01, ***p<0.001.

In order to explore the role of galectin-1 identified by proteomic approach in drug resistance, stable clone of galectin-1 overexpressing cells, K562/Gal-1, was constructed and characterized by q-PCR and immunoblotting. Compared with K562/pcDNA3.1(-) (K562/pc) cells transfected with the control vector, pronounced enhancement of galectin-1 expression at both transcriptional and translational levels was observed in K562/Gal-1 cells (Figure 5A and 5B). Further MTT assay results showed that overexpression of galectin-1 increased resistance to the detected chemical agents, with 4-fold higher IC_50_ of K562/Gal-1 cells (1.676±0.145 μg/ml of adriamycin and 0.84±0.087 μM of imatinib) than that of K562/pc cells (0.485±0.03 μg/ml of adriamycin and 0.23±0.035 μM of imatinib) (Table 3).

**Table 3:** Effects of galectin-1 and P38 inhibitor on the IC_50_ of adriamycin and imatinib in K562, K562/Gal-1, and K562/ADM cells

IC_50_	K562/pc	K562/Gal-1	K562/ADM					
	–	–	DMSO	SB202190	sc siRNA	Gal-1 siRNA	DMSO	SB202190
ADM(μg/ml)	0.485±0.03	1.676±0.145	1.636±0.141	1.29±0.125	25.56±0.41	14.76±0.45	26.51±0.44	16.24±0.48
imatinib(μM)	0.23±0.035	0.84±0.087	0.8±0.083	0.4±0.037	2.5±0.214	1.1±0.12	2.27±0.211	0.5±0.46

We next evaluated the effects of galectin-1 siRNA targeting treatment on the chemoresistance in K562/ADM cells. As shown in Figure 5C and 5D, treatment of K562/ADM cells with 50 nM galectin-1 siRNA induced down-regulation of galectin-1 expression at the mRNA and protein levels. Knockdown of galectin-1 in K562/ADM cells could increase the chemosensitivity to both adriamycin and imatinib treatment, with the IC_50_ of adriamycin decreasing from 26.56±0.41 μg/ml to 14.76±0.45 μg/ml, and that of imatinib from 2.5±0.214 μM to 1.1±0.12 μM as present in Table 3.

### Galectin-1 is a modulator of MDR1

**Figure 6:**
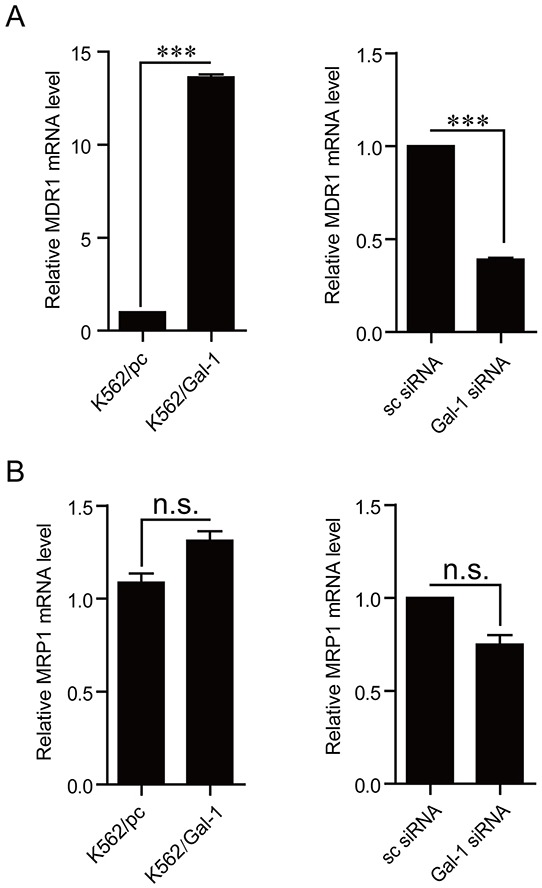
Galectin-1 modulates MDR1 expression The mRNA levels of MDR1 **A.** and MRP1 **B.** were detected in K562 cells stably overexpressing galectin-1 or in K562/ADM cells tranfected with Gal-1 siRNA for 48 h by q-PCR. The experiment was repeated for three times with similar results. ***p<0.001.

As shown by the above results, the trends of galectin-1 expression change are parallel to those of MDR1 in three types of cells with a gradual increase of resistance against adriamycin and imatinib. In the light of MDR1 directly contributing to the drug resistance by exporting drugs out of the cell, combined with our results showing that galectin-1 enhances the chemoresistance in CML, we proposed that galectin-1 may decrease the chemosensitivity via increase of MDR1 expression. To address this issue, q-PCR was applied to compare the mRNA levels of MDR1 between K562/pc and K562/Gal-1 cells, and between K562/ADM/sc siRNA and K562/ADM/Gal-1 siRNA. The results present in Figure 6A indicated that overexpression of galectin-1 up-regulated MDR1 transcription, while knockdown of galectin-1 decreased the mRNA levels of MDR1. Alteration of galectin-1 expression had little effect on the other well-studied drug transporter MRP1 (Figure 6B). The results suggest that augment of MDR1 expression involves in galectin-1-mediated chemoresistance.

### Galectin-1 induces MDR1 expression via P38 MAPK activation and NF-κB translocation

**Figure 7:**
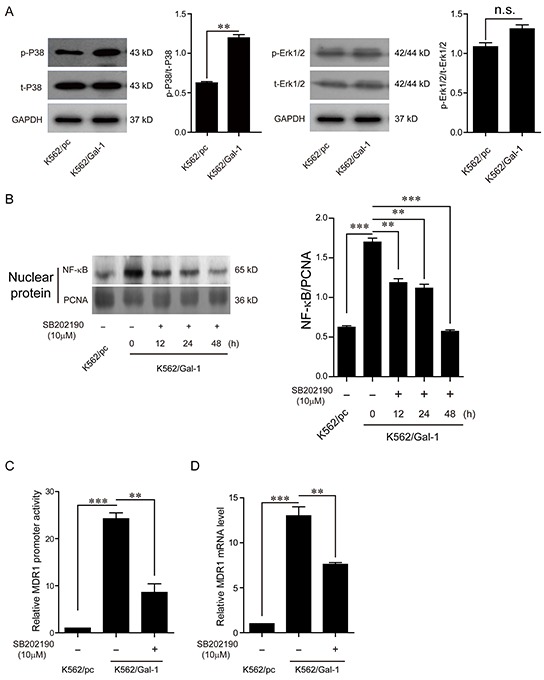
Galectin-1 induces MDR1 expression via P38 MAPK activation and NF-κB translocation **A.** K562/Gal-1 cells were pretreated with 10 μM P38 inhibitor SB202190 for 48 h prior to extraction of cell lysates for western blot analysis of the activation of Erk1/2 and P38. **B.** NF-κB translocation was determined by western blot analysis of NF-κB/p65 in nuclear extracts of K562/Gal-1 cells treated with P38 inhibitor SB202190 at the concentration of 10 μM for 12 h, 24 h, and 48 h. PCNA was used as the loading control. **C.** EGFP reporter assay was carried out to examine the MDR1 promoter transcriptional activity. K562/Gal-1 cells were pretreated with P38 inhibitor SB202190 for 1 h prior to transfection of the MDR1 promoter/EGFP reporter construct pGL3-proMDR1-EGFP or the control vector pGL3-EGFP. **D.** Inhibition of P38 activation triggered by galectin-1 decreased MDR1 expression. The mRNA levels of MDR1 were detected in K562/pc cells, K562/Gal-1 cells, and K562/Gal-1 cells treated with P38 inhibitor SB202190 for 1 h by q-PCR. Data are presented as the mean (±SD) of three independent experiments. **p<0.01, ***p<0.001.

It has been investigated that NF-κB transcription factor regulates MDR1 expression by specifically binding to an intronic response element of MDR1 gene promoter [16]. In the light of MAPK signal pathways mediating nuclear translocation of NF-κB, which is required for its transcription activity, and galectin-1 directly interacting with Ras, which subsequently activates Erk1/2 and P38 mitogen-activated protein kinase (MAPK) signal pathways [17-19], we proposed that the mechanisms of galectin-1 inducing MDR1 expression may involve MAPK signal activation and NF-κB translocation. In order to define the signal pathway participating in mediating galectin-1-induced NF-κB translocation, and subsequent MDR1 transcription, we first detected the effects of galectin-1 on the activation of Erk1/2 and P38 MAPK cascades. As shown in Figure 7A, overexpression of galectin-1 increased the phosphorylation levels of P38 rather than Erk1/2, implying that activation of P38 MAPK signal pathway may be responsible for mediating galectin-1 functions in K562 cells. Further suppression of P38 activation with the P38 inhibitor SB202190 attenuated NF-κB translocation (Figure 7B), MDR1 gene promoter activity (Figure 7C), and MDR1 mRNA levels (Figure 7D). Moreover, blockade of P38 activation in K562/Gal-1 cells decreased the chemoresistance to both adriamycin and imatinib treatment, with the IC_50_ of adriamycin reducing from 1.636±0.141 μg/ml to 1.29±0.125 μg/ml, and that of imatinib from 0.8±0.083 μM to 0.4±0.037 μM. Similar results were found in K562/ADM cells treated with SB202190 (Table 3). Combined with the above results, we suggest that galectin-1/P38 MAPK/NF-κB/MDR1 axis at least in part contributes to developing the chemoresistance in CML.

## DISCUSSION

Imatinib has been proved to be an efficient target drug with few adverse consequences for CML treatment. However, patients initially displaying positive response to imatinib become refractory to imatinib treatment due to the development of drug resistance. It has been found that MDR1 expression was more frequent in both advanced CML and acute myeloid leukemia (AML) patients [20, 21]. Mahon F et al. reported overexpression of *ABCB1* gene in leukemia cells results in resistance to imatinib [14]. Therefore, besides *BCR-ABL* amplification and mutation, decreased intracellular levels of imatinib caused by MDR1 up-regulation is also considered as a major reason leading to imatinib resistance [15]. However, the mechanisms by which the leukemia cells increase the expression of this transmembrane export pump are still unknown. We proposed that differentially expressed proteins identified by comparison of the protein profiles among a model system of the leukemia cells with different MDR1 expression levels may provide a hint for revealing the mechanisms modulating the expression of MDR1. K562/ADM cells selected by adriamycin exposure produce more abundant MDR1 than the parental K562 cells. Cultivation of K562/ADM cells in the condition without adriamycin selection for three consecutive months led to obtain the revertant K562/ADM cells with a moderate MDR1 expression. Coincident with Illmer T et al reports [15], a gradual augment of MDR1 in K562 cell lines is accompanied by an increase of resistant potentials against adriamycin and imatinib. By comparison of the protein profiles among three different K562 cell lines with a gradual increase of MDR1 expression, we identified galectin-1 as a candidate of MDR1 modulators for enhancing drug resistance. Further investigations revealed that galectin-1 induces MDR1 expression via P38 MAPK activation and NF-κB translocation, which contributes to the development of drug resistance.

Galectin-1 has a wide distribution in tissues and is modulated accompanying with the alteration of the physiological or pathological conditions. Accumulating evidences have demonstrated that galectin-1 is closely relevant to the tumor progression by promoting transformation, angiogenesis, and metastasis [22-25]. However, there are few reports exploring the role of galectin-1 in tumor chemoresistance. The endogenous expression levels of galectin-1 vary in different kinds of tumors. Some cancerous cells with high levels of endogenous galectin-1 expression, such as non-small cell lung cancer (NSCLC) cells, have relative high potentials of anti-chemotherapy. Suppression of galectin-1 sensitizes the NSCLC cells to platinum-based chemotherapy [19]. In our studies, we found the expression levels of the endogenous galectin-1 are pretty low in K562 cells, whereas significant up-regulation of galectin-1 is observed in the corresponding chemoresistant K562/ADM cells. Moreover, treatment of K562 cells with adriamycin or imatinib could increase galectin-1 expression, suggesting that tumor cells may gradually develop drug resistance along with the chemotherapy. These results imply that galectin-1 might be a potential biomarker for the chemoresistant ability of tumor cells, and provide a novel target for combined therapy for enhancing the efficiency of the chemical drugs.

Promotion of apoptosis by galectin-1 in the activated T cells has been extensively investigated in the past two decades [26, 27]. The molecular mechanisms underlying galectin-1 inducing apoptosis involve suppression of anti-apoptotic protein expression and stimulation of caspases [28]. As one mechanism by which tumor cells develop drug resistance is associated with resistance to apoptosis, it seems paradoxical that galectin-1 on one hand induces apoptosis, and on the other hand contributes to drug resistance. We initially presumed that galectin-1 may exert an oppose effect on the apoptosis in K562 cells as compared to the activated T cells. Therefore, flow cytometric analysis was applied to determine the effects of galectin-1 on the apoptosis of K562 cells. Contrary to our speculation, galectin-1 significantly promoted apoptosis of K562 cells treated with or without adriamycin (data not shown). It seems that apoptosis induced by galectin-1 contributes little to the galectin-1-triggered drug resistance. In the light of reports suggesting that galectin-1 does not activate a full apoptotic pathway in some kinds of cells including T cells [29], T leukemia cells, promyelocytic cells and activated neutrophils [30], the possible explanation may be that K562 cells induced by galectin-1 undergo partial apoptotic process, which has less relevance with drug resistance than the full apoptotic process does. However, further investigations are required to clarify the relationship between galectin-1-elicited apoptosis and drug resistance.

In summary, we found that galectin-1 was a novel modulator of MDR1 by proteomic analysis of a model system of leukemia cell lines with a gradual increase of MDR1 expression and drug resistance, and NF-κB translocation induced by P38 MAPK activation was responsible for the enhancing effects of galectin-1 on MDR1, suggesting that galectin-1 might be a novel target for improving the efficacy of CML chemotherapy.

## MATERIALS AND METHODS

### Reagents

The cell proliferation assay reagent methylthiazoletetrazolium (MTT) was purchased from Roche (Mannheim, Germany). RMPI-1640 and fetal bovine serum (FBS) were obtained from GIBCO (Carlsbad, CA, USA). Adriamycin (ADM) was purchased from Sangon Biotech (Shanghai, China). Imatinib was purchased from Selleckchem (Houston, Texas, USA). The polyvinylidenedifluoride (PVDF) membrane was from Millipore (Billerica, MA, USA). The enhanced chemiluminescence (ECL) detection kit was obtained from Pierce (Rockford, IL, USA). Anti-phospho-Erk1/2 (cat no. 4370), anti-Erk1/2 (cat no. 4695), anti-phospho-P38 (cat no. 4511), anti-P38 (cat no. 8690), anti-PCNA (cat no. 13110), anti-GAPDH (cat no. 2118), anti-NF-κB/p65 (cat no. 8242), anti-Galectin-1/LGALS1 (cat no. 1293) antibodies, P38 inhibitor SB202190, anti-rabbit IgG, HRP-linked antibody (cat no. 7074), and anti-mouse IgG, HRP-linked antibody (cat no. 7076) were obtained from Cell Signaling Technology (Danvers, MA, USA). IPG strips (pH 3-10 nonlinear), SDS, acrylamide, methylene bisacrylamide, TEMED, CHAPS, Bio-Lyte 3-10 ampholyte 40% solution, Tris, glycine, and iodoacetamide were from Bio-Rad (Hercules, CA, USA). PureZOL RNA, First-Strand cDNA synthesis kit and SYBR green q-PCR kit were purchased from Bio-Rad. *HindIII*, *BamHI*, *NcoI*, *XbaI* and T4 ligase were purchased from Thermo Fisher (Waltham, MA, USA).

### Cell culture

A model system of leukemia cell lines including K562 cells and the adriamycin resistant cells, K562/ADM, were gifts from Professor Yifei Wang in Department of cellular biology of Jinan University (Guangzhou, China). The revertant K562/ADM cells were obtained by culture of K562/ADM cells without adriamycin for three consecutive months. K562 and the revertant K562/ADM cells were maintained in RPMI-1640 supplemented with 10% FBS and 10 mM HEPES (Sigma) buffer at 37°C in an atmosphere of 5% CO_2_. K562/ADM cells were cultured in the complete RPMI-1640 media containing 500 ng/ml adriamycin.

### Cell viability assay

Cells were seeded into 96-well culture plates at a density of 1×10^4^ cells per well. After 4 h, cells were treated with serially diluted adriamycin (0.05, 0.1, 0.2, 0.4, 0.8, 1.6, 3.2 μg/ml for K562, K562/pc and K562/Gal-1 cells; 1.6, 3.2, 6.4, 12.8, 25.6, 51.2, 102.4 μg/ml for K562/ADM, the revertant K562/ADM, K562/ADM/sc siRNA and K562/ADM/Gal-1 siRNA cells), or imatinib (0.05, 0.1, 0.2, 0.4, 0.8 μM for K562, K562/pc and K562/Gal-1 cells; 0.4, 0.8, 1.6, 3.2, 6.4, 12.8, 25.6 μM for K562/ADM, the revertant K562/ADM, K562/ADM/sc siRNA and K562/ADM/Gal-1 siRNA) for 48 h. The viability of cells was determined by the MTT assay as previously described [31].

### Sample preparation for 2D-PAGE

K562, K562/ADM and the revertant K562/ADM cells cultured in 75 cm^2^ flask were harvested and lysed in 150 μl lysis buffer containing 7 M urea, 2 M thiourea, 4% CHAPS, 65 mM DTT, 0.2% Bio-Lyte 3-10 ampholyte, 50 μg/ml RNase A, 200 μg/ml DNase I, and protease inhibitor cocktail. Samples were incubated at room temperature for 10 min, kept on ice for 2 h, and centrifuged at 12,000 g for 30 min at 4°C. The supernatant was collected and the protein concentrations were detected using the Bradford method.

### 2D-PAGE and image analysis

An equal amount (1 mg) of protein sample was mixed with rehydration buffer complemented with 7 M urea, 2 M thiourea, 4% CHAPS, 65 mM DTT, 0.2% Bio-Lyte 3-10 ampholyte and 0.001% bromophenol blue, and loaded on a 17 cm, pH3-10 nonlinear immobilized pH gradient (IPG) gel strip (Bio-Rad). The IPG strips were then passively rehydrated at 20°C for 13 h, and subjected to isoelectric focusing (IEF) performed at 20°C at 100 V for 30 min, 150 V for 3 h, 250 V for 1 h, 500 V for 1 h, 1000 V for 2 h, 5000 V for 3 h, 8000 V for 64000 V/h, and 500 V for 24 h. After IEF separation, the strip was equilibrated, and separated on 12% SDS-PAGE gels as previously described [32]. The gel was stained with Coomassie brilliant blue G250 (Bio-Rad), and scanned with a UMAX POWERLOOK 2100XL USB scanner (UMAX, Dallas, TX, USA). PDQuest 8.0 software (Bio-Rad) was used to analyze the images.

### Mass spectrometric analysis and database search

In-gel digestion of the differentially expressed proteins was carried out before mass spectrometric analysis. Briefly, the protein spots were excised, destained in 25 mM ammonium bicarbonate 50% NH_4_HCO_3_/acetonitrile (ACN) (v/v), dehydrated in 100% ACN, and then incubated with trypsin at 37°C overnight. The peptides were extracted, dried in a vacuum concentrator for 3 h, and subjected to tandem time-of-flight mass spectrometry (ABI 4800 TOF-TOF) analysis. Mascot software (Matrix science, London, UK) was applied to search IPI (International Protein Index) databases for protein identification.

### Construction of K562/Gal-1 cell line stably overexpressing galectin-1

The full-length galectin-1 coding sequence was amplified from cDNA synthesized by reverse transcriptase polymerase chain reaction (RT-PCR) using the total RNA extracted from K562/ADM cells as the template. Briefly, cDNA was synthesized via reverse transcription using the oligo dT_18_, and subjected to PCR amplification of the full-length galectin-1 coding sequence (NCBI accession no. NM_002305.3). The PCR procedure comprised of an initial step at 94°C for 5 min, followed by 40 cycles of 94°C for 30 s, 58°C for 15 s, and 72°C for 1 min, and an additional extension at 72°C for 30 min. The following primers were used for galectin-1 amplification: 5′-AGCGGATCCATGGCTTGTGGTCTGGTC-3′ (forward, *BamHI* site underlined) and 5′-TATAAGCTTTCAGTCAAAGGCCACACA-3′ (reverse, *HindIII* site underlined). The PCR products were purified by the TIANGEN purification kit (TIANGEN, Beijing, China), digested with *BamHI* and *HindIII*, and inserted into the pcDNA3.1(-) vector (Invitrogen, Carlsbad, CA, USA) to obtain the recombinant plasmid pcDNA3.1(-)/Gal-1, which was subsequently sent to Sangon Company (Shanghai, China) for sequencing.

The recombinant plasmid pcDNA3.1(-)/Gal-1 harboring the correct galectin-1 coding sequence and the control vector pcDNA3.1(-) were transfected into K562 cells with lipofectamine LTX (Invitrogen) following the manufacturer's protocol. Briefly, 3×10^4^ K562 cells seeded in an individual well of a 24-well culture plate were transfected with 1 μg of plasmid DNA. After transfection for 48 h, cells were selected in complete RPMI-1640 media containing G418 (500 μg/ml) for 2 weeks, and subsequently replated (10 cells/well) in 96-well culture plate for continuous G418 selection. After cultured for 10 days, individual G418-resisitant colonies were picked, propagated and screened for K562/Gal-1 cell clone stably expressing galectin-1 by q-PCR and western blotting. The sequences of galectin-1 and GAPDH primers for q-PCR were shown in Table 4. GAPDH was used as an internal control.

**Table 4:** The sequences of primers for real time quantitative PCR

Gene name	Accession No.	forward (5′-3′)	reverse (5′-3′)	product length (bp)
Gal-1	NM_002305	CGAGTGCGAGGCGAGGTG	CGTTGAAGCGAGGGTTGAAGTG	100
MDR1 (ABCB1/Pgp)	NM_000927.4	TTGCCTATGGAGACAACAGCC	ACGAGCTATGGCAATGCGTT	173
MRP1 (ABCC1)	NM_004996.3	CTACCTCCTGTGGCTGAATCTG	CATCAGCTTGATCCGATTGTC	151
GAPDH	NM_002046.5	CCCACTCCTCCACCTTTGAC	TCTTCCTCTTGTGCTCTTGC	182

### Suppression of galectin-1 expression by small interfering RNA

A small interfering RNA (siRNA) targeting galectin-1 (Gal-1 siRNA) [19] with the sequence of sense 5′-GCUGCCAGAUGGAUACGAAUUdtdt-3′, and anti-sense 5′-AAUUCGUAUCCAUCUGGCAGCdtdt′ was synthesized by RiboBio (Guangzhou, China). A scrambled siRNA (sc siRNA) obtained from RiboBio was used as a negative control. For siRNA-mediated inhibition of galectin-1 gene expression, K562/ADM cells were transfected with Gal-1 siRNA or sc siRNA at a final concentration of 50 nM using RNA MAX siRNA Transfection Reagent (Invitrogen) according to the manufacturer's instructions. Silencing efficiency was estimated at mRNA and protein levels by q-PCR and western blotting.

### Quantitative PCR analysis

Total RNA was isolated with PureZOL according to the manufacturer's instructions. A first-strand cDNA synthesis kit was applied to produce cDNAs from 1μg of total RNA, which were then used as templates for q-PCR amplification with the SYBR green q-PCR Kit using the CFX96 Touch™ Real-Time PCR Detection System. The primers of galectin-1, MDR1, MRP1 and GAPDH were showed in Table 4. GAPDH was amplified as an internal control. The q-PCR conditions were 94°C for 5 min followed by 40 cycles of 95°C for 5 s, 59°C for 20 s.

### Western blotting analysis

Cells were harvested and lysed in 1 × SDS-PAGE loading buffer, then centrifuged at 12,000 g for 30 min at 4°C to remove the insoluble components. The resultant protein samples were resolved by 10% SDS-PAGE gel and transferred to a PVDF membrane. The membrane was blocked at room temperature for 1 h in TBST (25 mM Tris, pH 7.4, 150 mM NaCl, and 0.1% Tween-20) containing 5% non-fat dry milk, and subsequently incubated with the primary antibodies at 4°C overnight followed by incubation with goat anti-rabbit or rabbit anti-mouse IgG, HRP-linked antibody for 1 h at room temperature. The blots were detected with an ECL detection kit (Pierce) according to the manufacturer's procedure. GAPDH was used as the reference control. The results were analyzed by Quantity One software to determine the ratio relative to GAPDH.

### Extraction of nuclear proteins

Cells were seeded in 6-well culture plates at a density of 2.5×10^5^ cells per well. After cultivation for 6 h, K562/Gal-1 cells were treated with P38 inhibitor SB202190 at the concentration of 10 μM for 12 h, 24 h, and 48 h before extraction of the nuclear proteins using the Nuclear and Cytoplasmic Protein Extraction Kit (KeyGEN Biotech, Nanjing, China) according to the manufacturer's instructions. The protein concentration was determined by a BCA protein assay kit (Pierce). The nuclear proteins (NF-κB/p65 and PCNA) were analyzed by western blotting.

### Promoter activity reporter assay

A promoter/EGFP reporter plasmid containing the MDR1 promoter region (-982 to -7) was constructed by replacement of the luciferase reporter with EGFP reporter in pGL3-basic (Promega; Madison, WI, USA). Briefly, EGFP reporter was first amplified by PCR using the plasmid EGFP-N3 as a template and the primer pair: 5′-GCCACCATGGTGAGCAAGGGCGAG-3′ forward, and 5′-CGCGTCTAGATTACTTGTACAGCTCGTCCATGCCGAGAG-3′ reverse, (*NcoI* and *XbaI* sites were underlined, respectively), and substituted for the luciferase reporter in the plasmid pGL3-basic at the identical sites to obtain the EGFP reporter plasmid pGL3-EGFP. MDR1 promoter region was then amplified from human genomic DNA extracted from HUVEC cells using the following primer pair: 5′-ATATAAGCTTCTGCAGGGGCTTTCCTGTG-3′ and 5′-TATAAAGCTTCTGCAGAAAAATTTCTCCTAGCC-3′ (*HindIII* site was underlined), and inserted into pGL3-EGFP at the identical site to construct a MDR1 promoter/EGFP reporter plasmid pGL3-proMDR1-EGFP.

Cells were seeded into 12-well tissue-culture plate with 5 × 10^5^ each well. After 6 h, K562/Gal-1 cells were pretreated with P38 inhibitor SB202190 for 1 h prior to transfection. Cells were transfected with the MDR1 promoter/EGFP reporter construct pGL3-proMDR1-EGFP or the control vector pGL3-EGFP using lipofectamine LTX (Invitrogen) according to the manufacturer's instruction. After 48 h transfection, cells were harvested, washed three times with cold PBS, and subjected to analysis using a BD Bioscience FACScan (Becton Dickinson, NJ, USA). Data were analyzed and presented using the Cell Quest software. The relative fluorescence intensity was calculated by dividing the fluorescence intensity of cells transfected with pGL3-proMDR1-EGFP by that of cells transfected with the control vector pGL3-EGFP.

### Statistical analysis

The statistical analyses were performed using GraphPad Prism software 5.01. The student's t-test was used to compare data between two groups, and one way ANOVA followed by Tukey's multiple comparison test was used for multiple comparison data. Differences were considered significant at *p< 0.05, **p< 0.01, and ***p< 0.001.
